# In-silico enhanced animal study of pulmonary artery pressure sensors: assessing hemodynamics using computational fluid dynamics

**DOI:** 10.3389/fcvm.2023.1193209

**Published:** 2023-09-07

**Authors:** Jan Brüning, Pavlo Yevtushenko, Adriano Schlief, Tobias Jochum, Livia van Gijzen, Sonja Meine, Jan Romberg, Titus Kuehne, Andreas Arndt, Leonid Goubergrits

**Affiliations:** ^1^ Institute of Computer-assisted Cardiovascular Medicine, Deutsches Herzzentrum der Charité, Berlin, Germany; ^2^ Charité - Universitätsmedizin Berlin, Berlin, Germany; ^3^ Biotronik, Berlin, Germany; ^4^ Einstein Center Digital Future, Berlin, Germany

**Keywords:** heart failure, pulmonary artery, pressure sensor, computational fluid dynamics, wall shear stress, oscillating shear index, computed tomography, porcine animal model

## Abstract

To assess whether in-silico models can be used to predict the risk of thrombus formation in pulmonary artery pressure sensors (PAPS), a chronic animal study using pigs was conducted. Computed tomography (CT) data was acquired before and immediately after implantation, as well as one and three months after the implantation. Devices were implanted into 10 pigs, each one in the left and right pulmonary artery (PA), to reduce the required number of animal experiments. The implantation procedure aimed at facilitating optimal and non-optimal positioning of the devices to increase chances of thrombus formation. Eight devices were positioned non-optimally. Three devices were positioned in the main PA instead of the left and right PA. Pre-interventional PA geometries were reconstructed from the respective CT images, and the devices were virtually implanted at the exact sites and orientations indicated by the follow-up CT after one month. Transient intra-arterial hemodynamics were calculated using computational fluid dynamics. Volume flow rates were modelled specifically matching the animals body weights. Wall shear stresses (WSS) and oscillatory shear indices (OSI) before and after device implantation were compared. Simulations revealed no relevant changes in any investigated hemodynamic parameters due to device implantation. Even in cases, where devices were implanted in a non-optimal manner, no marked differences in hemodynamic parameters compared to devices implanted in an optimal position were found. Before implantation time and surface-averaged WSS was 2.35±0.47 Pa, whereas OSI was 0.08±0.17, respectively. Areas affected by low WSS magnitudes were 2.5±2.7 cm${}^{2}$, whereas the areas affected by high OSI were 18.1±6.3 cm${}^{2}$. After device implantation, WSS and OSI were 2.45±0.49 Pa and 0.08±0.16, respectively. Surface areas affected by low WSS and high OSI were 2.9±2.7 cm${}^{2}$, and 18.4±6.1 cm${}^{2}$, respectively. This in-silico study indicates that no clinically relevant differences in intra-arterial hemodynamics are occurring after device implantation, even at non-optimal positioning of the sensor. Simultaneously, no embolic events were observed, suggesting that the risk for thrombus formation after device implantation is low and independent of the sensor position.

## 1. Introduction

Heart failure (HF) is a leading cause of death and hospital admission (1) with high overall prevalence of 1–2%. This prevalence is expected to increase significantly, especially in the aging industrialized nations. HF is a complex disease that can have several causes, co-morbidities, and sequelae (2). As heterogeneous as the disease are the therapeutic options that range from close monitoring to invasive surgery for treating underlying problems such as heart valve diseases. While the lifetime management and therapeutic option for heart failure are steadily improving, outcomes of patients after hospitalization are still poor and methods for reduction of hospital readmissions are an ongoing focus of research in HF (3). Here, a promising approach is telemonitoring, which was shown to significantly reduce mortality and readmission (4). In addition, there are biomarkers, such as the pulmonary artery pressure (PAP), allowing early prediction of worsening of HF, as for example acute decompensation. However, the PAP is usually only assessed during intensive care stays as its measurement requires invasive catheterization.

To overcome this limitation and allow assessment of the PAP in telemonitoring settings, a new class of implantable medical devices was introduced relatively recently: the pulmonary artery pressure sensor (PAPS) (5). These devices are implanted into the pulmonary artery using a catheter and improve monitoring of HF patients, aiming for early detection of acute decompensation, that can ideally be mitigated using pharmaceutical therapies to avoid readmission of these patients (6).

Currently, the CardioMEMS HF-System (Abbott) (7, 8) is the only device available in clinical routine. Another device under development, the Cordella HF system (Endotronix, Inc) (9, 10), is currently undergoing clinical evaluation. The systems differ in size and recommended implantation site. While the dimensions of the CardioMEMS are 15.0mm×3.4mm×2.0mm in length, width, and height, respectively, the Cordella system is slightly longer and wider with dimensions of 19.3mm×3.8mm×1.9mm. The preferred implantation site for the CardioMEMS are inferior and lateral branches of the left pulmonary artery (LPA), whereas the Cordella system is to be implanted in the right pulmonary artery (RPA), where the interlobar artery typically turns downwards and posterior. Even though the devices are comparable in size, the recommended vessel diameters of the implantation differ markedly and are 7–11 mm for the CardioMEMS and 12–26 mm for the Cordella system, respectively. According to clinical trials, both devices facilitate significant reduction in readmission and hospitalization of up to 60 percent and thus improvement of HF management (11, 12).

A novel PAPS device with dimensions similar to those of the previously mentioned systems, that is to be implanted into either left or right pulmonary artery with target vessel diameters of 9–14 mm is currently under development. To demonstrate and guarantee device safety and efficacy of any novel medical device, preclinical evaluation in frames of bench tests and animal experiments are necessary according to relevant regulations, such as the European medical device regulation. Such an animal experiment was conducted for the novel device, aiming to address various aspects of its safety and efficacy including the feasibility and safety of the implantation procedure, device fixation, durability, function, and to assess the risk of device-related complications.

To this day, animal experiments are still required for evaluation of medical devices. However, they are affected by several limitations with respect to the information and parameters that can be assessed and their translation towards use in humans is often challenging. In addition, the ethical burden of animal experiments is high, and they are cost- and time-intensive. Therefore, research on alternative methods is ever increasing. One alternative to animal experimentation arises from in-silico modelling. These methods hold the promise to model different aspects of healthy as well as pathologic systems even in a personalized manner (13). They are also widely used in medical device research and are becoming increasingly important for evaluation and certification of novel devices (14).

Similarly, the device safety and efficacy of the novel PAPS device is to be evaluated using in-silico models. In frames of the Horizon 2020 Research and Innovation Action SIMCor (www.simcor-h2020.eu), a modelling pipeline is to be elaborated and validated, that allows prediction of three clinical endpoints for PAPS devices. These endpoints are device migration, perforation of the pulmonary artery by the device fixation, and device-related thrombosis. In order to generate data for parameterization and validation of the models that are developed for prediction of these endpoints, chronic animal experiments were conducted. However, to maximise the use of the animal experiments with respect to the 3R principles, other aspects, such as evaluation and testing of the device implantation procedure, accuracy of pressure measurements, and data transmission from device to a monitoring system were evaluated as well. Thus, the animal experiments were not only intended for assessing device safety and efficacy, as well as the impact of the device on the intra-arterial hemodynamics, but to provide data for validation of models used for assessing these aspects of the novel sensor in in-silico clinical trials.

This study describes an approach for calculation of the intra-arterial hemodynamics assessing parameters associated with the third clinical endpoint of device thrombosis. Information on the intra-arterial hemodynamics before and after device implantation are important to assess and understand potential risks for thrombus formation, which can be caused by disturbed flow conditions resulting from the implant’s interaction with the blood flow (15). This information cannot be assessed in-vivo. While magnetic resonance imaging-based methods for measurement of in-vivo hemodynamics exist (16), imaging artefacts caused by the metallic implants render this method unavailable. Furthermore, magnetic resonance (MRI) imaging compatibility of the device must be assessed first.

Therefore, the immediate aim of this study was to support in-vivo animal experiments by using in-silico models aiming to enhance the information that can be gathered from these experiments. To facilitate this, information on the intra-arterial hemodynamics before and after implantation of the device were modelled using computational fluid dynamics (CFD). Computed tomography (CT) data allowed to assess the subject-specific information on the pulmonary artery (PA) geometry as well as the implantation sites of the devices. Surface geometries of the PA were reconstructed and sensors were virtually implanted, resulting in virtual twins of each animal investigated within the in-vivo experiments. Subsequently, different hemodynamic parameters associated with thrombus formation were calculated and compared.

## 2. Materials and methods

### 2.1. Animal experiments

The chronic animal experiments were conducted at the animal research facilities at the Charité - Universitätsmedizin Berlin from April until October 2022. They were approved by the ethics committee of the responsible veterinary department for animal protection at the relevant competent authority, the Regional Office for Health and Social Affairs Berlin (registration number G 0091/21). The animals were treated, fed and cared for according to the guidelines of the European and German Society for Laboratory Animal Science (FELASA, GV-SOLAS), as well as standard operating procedures established at the animal research facilities.

Device implantation was performed in 10 pigs, with an approximate weight of 60 kg at the day of device implantation. For each animal CT acquisition was performed seven days before and immediately after device implantation, as well as at 30 and approximately 60 days after implantation, resulting in 4 acquisitions per animal. Two sensors were implanted into each of the 10 animals, one into the left and right pulmonary artery, respectively. Thus, the number of required animals could be halved. This approach was chosen as the hemodynamic interaction between the sensors implanted into each side of the pulmonary artery are neglectable. One animal died after implantation.

During the animal experiments following steps were conducted for each animal following the general procedure: (1) sedation and anaesthesia, (2) pre-treatment CT, (3) device implantation including establishing a venous access, guidewire placement with PA visualization via angiography, implantation site identification and finally sensor implantation followed by (4) post-treatment as well as follow-up CTs, and finally (5) euthanasia and device explantation. For general anesthesia, which was performed in total four times, the animals were sedated with a mixture of atropine, ketamine, xylazine, and midazolam and were intubated, if necessary, with propofol administration and connected to inhalation anaesthesia (low dosis isoflurane 0.4–1% and oxygen) to maintain anaesthesia. In addition, the animals received midazolam via a perfusor (0.1 mg/kg/h). For analgesia, they also received fentanyl (1–4 μg/kg/h) via a perfusor as well as heparin diluted in NaCl via a continous infusion rate of 2 mL/h. To avoid infection they recieved intra-venous antibiotics. The implants were placed using jugular access (via vena jugularis externa). The access was established via a 21 F sheath by venae sectio. The 21 F sheath was a prototype developed for the PAPS. First, a 12 F introducer was used to widen the vein, followed by the 21 F sheath. As the main focus of the experiments was to obtain information for subsequent validation of models for device implantation and device effect simulation, devices were not only placed in the ideal arterial diameter range of 11–14 mm, but also in proximal parts of the left and right pulmonary artery. This approach was chosen to increase the number of occurrences of device migration and thus hemodynamic disturbances that might cause device thrombosis. Respectively, implanted devices were later subdivided into the optimally and sub-optimally implanted devices for further analysis. Euthanasia was performed by injection at least 60 mL KCL under general anaesthesia.

CT image data was acquired using a dual-source multi-slice spiral CT scanner (SOMATOM Definition Flash, Siemens Healthineers, Erlangen, Germany) with a tube voltage of 100 kV, an in-plane resolution of 0.67mm×0.67mm, and a slice-thickness of 0.7 mm. Acquisitions were triggered using electrocardiographic information to facilitate averaging over several heartbeats and reconstructions of temporally resolved images at a temporal resolution of 10 phases per heartbeat. Contrast agents were administered to better visualize the blood pool (Imeron 300, 2–5 mL/kg).

### 2.2. CT image data post-processing

CT image data was used to reconstruct the end-diastolic 3D geometry of the PA including main, left and right PA. The entire pulmonary artery in the field of view was reconstructed. During image acquisition, the aim was to assess at least 80 mm of length of the left and right PA, to ensure that the targeted implantation site, which features relatively small diameters and can be located far downstream to the main bifurcation, was included in the field of view. The reconstruction was performed using ZIBAmira (v. 2015.28, Zuse Institute Berlin, Germany). Mostly manual procedures and few semi-automatic methods were used to reconstruct the 3D anatomy of the PA. In general, all image voxels above a specific Hounsfield Unit (HU) threshold were considered to be potential candidates of the PA lumen. No fixed threshold could be defined for all 40 data sets, as high variations in the contrast agent concentration were observed. Overall, the individual HU thresholds chosen for reconstruction varied between 100 and 250. The PA lumen was reconstructed slice by slice, beginning from the right ventricular outflow tract (RVOT), using different tools implemented in ZIBAmira, such as brushes, flood fill, as well as region-grown algorithms. The reconstruction was corrected by slicing through the data stack in all three directions. The voxel label field was then used to generate initial triangulated surface meshes, which were subsequently smoothed. Finally, centrelines were generated and used to automatically calculate major geometric parameters, such as the length and mean diameters of vessel segments of the main, left and right pulmonary artery, as well as the bifurcation angle between LPA and RPA.

### 2.3. Virtual PAPS implantation procedure

**Figure 1:**
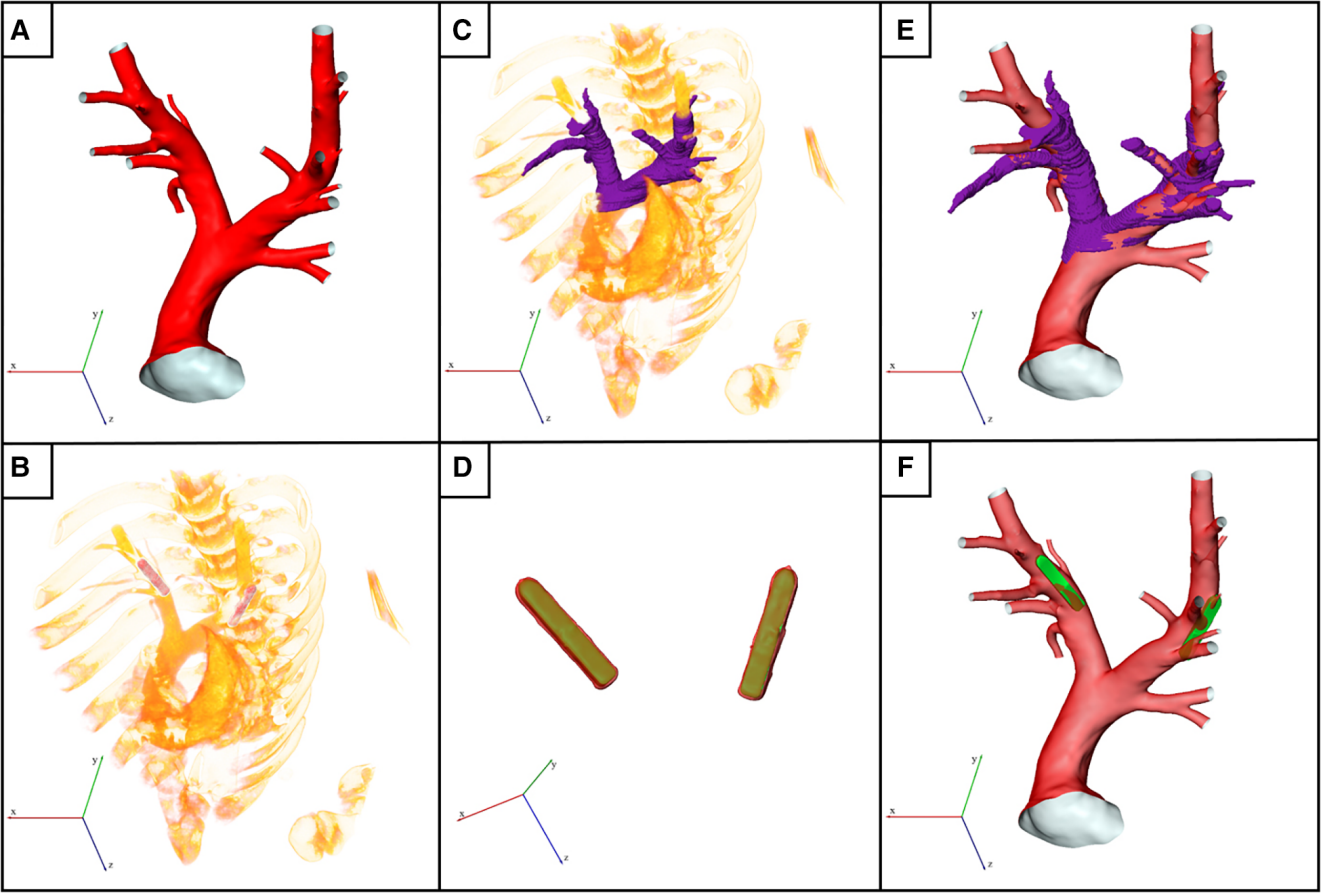
Illustration of the virtual device implantation procedure. (**A**) Reconstructed surface of an exemplary pulmonary artery (PA) acquired before pulmonary artery pressure sensor (PAPS) implantation. (**B**) Volume rendering of the post-interventional 3D computed tomography (CT) data showing both implanted PAPS. (**C**) Partial reconstruction of the post-interventional PA used for subsequent registration with pre-interventional dataset. (**D**) Reconstruction of both PAPS using high Hounsfield unit thresholds (red colored surface) was used to register the PAPS 3D model (green surface) to the exact post-interventional position. (**E**) Registered surfaces of pre- and post-interventional datasets. (**F**) Final geometry used for computational fluid dynamics simulation, generated by virtually implanting the PAPS.

For simulation of the hemodynamics after device implantation, the PAPS devices had to be virtually implanted into the reconstructed PA geometries while ensuring that the device position mimics that of the real post-implantation situation. First, the PA geometry that was reconstructed from pre-interventional CT following the description of the previous section was used as baseline (see Figure 1A). Subsequently, the CT data acquired 30 days after implantation was assessed to identify the subject-specific location of the sensors (Figure 1B). The scan 30 days after implantation was used instead of the immediate post-procedural CT, as the animals did not awake and move before the latter one, meaning that device dislocation due to the animals’ movement could not yet have occurred. 3D geometries of the sensor body provided by the manufacturer were then virtually implanted into the PA.

First, the two CT data sets had to be registered, to account for differences in the animal position and scanner coordinates. To facilitate this, a partial reconstruction of the PA bifurcation region was performed (Figure 1C). Then, the sensor bodies were automatically reconstructed in the post-interventional CT data set using (Figure 1D, red) a high HU threshold of 1200. Subsequently, the sensor geometries are registered with these automatic reconstructions of the real sensor positions in the follow-up data, using ZIBAmira, by minimizing the root mean square of the distances between both geometries. Figure 1D shows the superposition of the PAPS geometries (yellow) and the image-based reconstructions of the implanted devices (red). Similarly, the pre-interventional surface geometry of the PA is registered with the rough PA reconstruction performed using the follow-up CT data, as shown in Figure 1E. In individual cases, slight corrections of the sensor position were necessary to align the sensor surface with the vessel wall and in order to compensate smaller changes in the PA geometry due to somatic growth, or caused by the implantation procedure.

Finally, the sensor geometries are positioned within the PA at the exact locations as indicated in the follow-up CT data (see Figure 1F). To obtain a connected surface geometry enclosing the entire blood pool, the sensor body is subtracted from the PA lumen using the Boolean domain operations provided by STAR-CCM+ (15.04, Siemens PLM, Plano, Texas). While the result is a joint fluid domain, separate surfaces for the sensor body and the PA surface are retained. This procedure was chosen over reconstruction of the PA geometry from the follow-up CT data, as the metallic sensor bodies caused artifacts that rendered reconstruction of the intricate details of the PA, especially smaller branching vessels, impossible. The fixation wires of the sensor were not considered in its geometric model.

### 2.4. Computational fluid dynamics analysis

**Figure 2:**
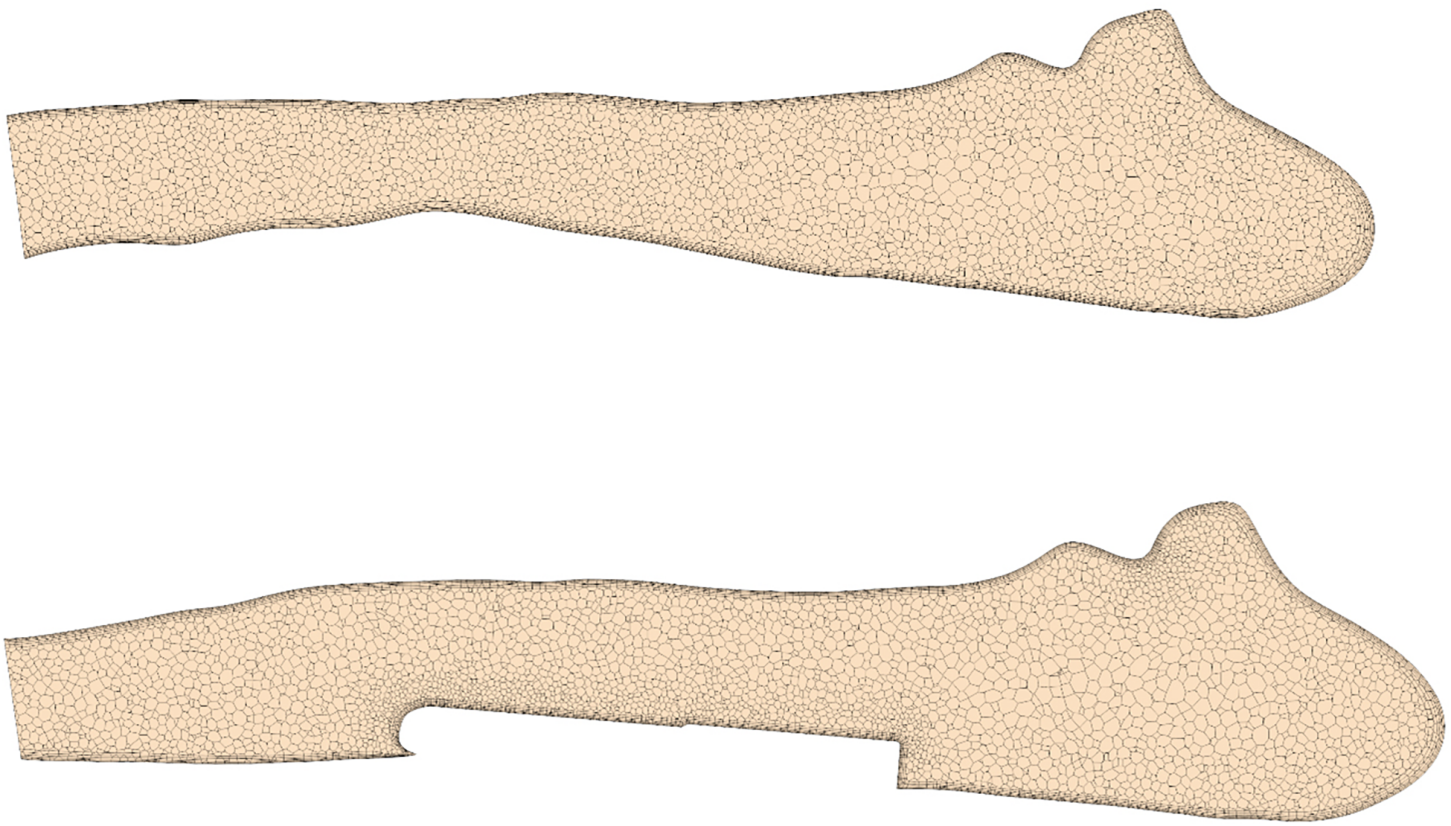
Cross-section of a pulmonary artery showing the numerical discretization with and without virtually implanted device.

Blood flow simulations were performed using STAR-CCM+ (15.04, Siemens PLM, Plano, Texas). The software provides both meshing algorithms to construct the computational mesh and finite-volume solvers for calculation of the intra-arterial hemodynamics. The fluid domain is discretized using polyhedral cells. In addition, six prism layers are introduced at at the vessel and sensor wall to resolve near-wall flows accurately. This information is required to accurately calculate hemodynamic parameters such as the wall shear stress (WSS, τ) and the oscillating shear index (OSI). Exemplary cross-sections of the numerical meshes are shown in Figure 2 for a configuration with and without implanted sensor body.

A mesh independence study found that meshes with approximately 1 million cells (2.5 million vertices) generated with a base size of 0.75 mm allows accurate calculation of OSI and time-averaged WSS (TAWSS) with errors below 3% compared to simulations with very fine meshes (4 million cells), while simultaneously reducing the computational costs (see Supplemental Material). Furthermore, the chosen cell size ensured a wall y+ at the order of one throughout the whole vessel/sensor wall.

Blood was modelled as an incompressible fluid with a density of 1050 kg/m${}^{3}$ and a shear-rate dependent viscosity following a Carreau-Yasuda model with coefficients described by Abraham et al. (17). Given the high Reynolds numbers (>2000) expected at peak systolic flow rates, a k-omega SST turbulence model is used to account for turbulent effects. The vessel wall was assumed to be rigid and a no-slip boundary condition was applied. Each simulation included two consecutive cardiac cycles with the second cycle being used for post-processing of results. Simulation of two cycles was considered sufficient to obtain converged results, as errors between OSI and TAWSS calculated in the second and fifth cycle were less then one percent (see Supplemental Material). A constant time step of Δt=1 ms was used for all simulations. At the main PA (MPA) inlet, a time dependent volumetric flow waveform with a constant velocity profile was prescribed while a constant pressure boundary condition was used at all outlets.

**Figure 3:**
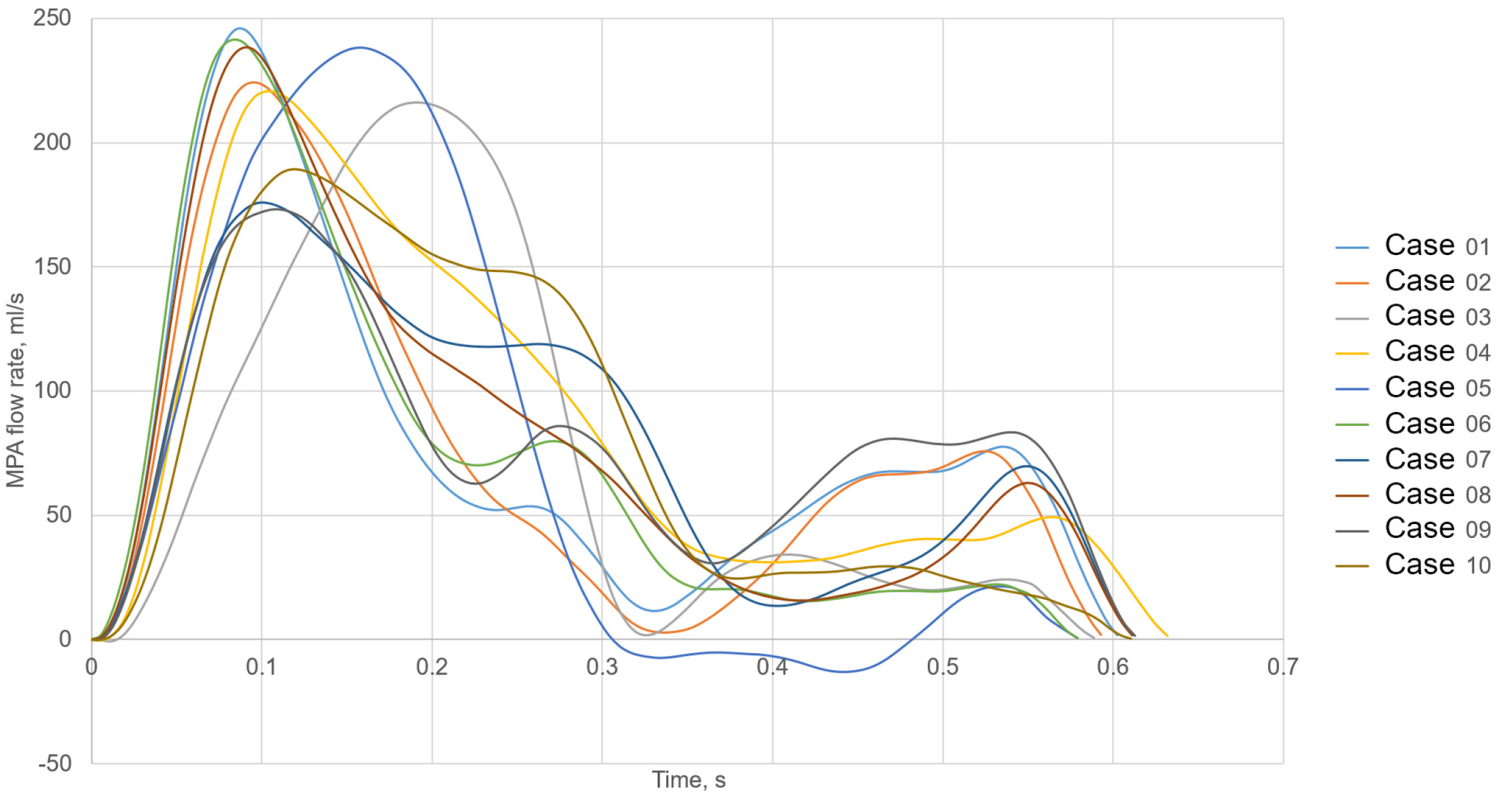
Volume flow waveforms used as the main pulmonary artery (MPA) inlet boundary condition for all 10 cases.

Furthermore, a constant low turbulence intensity of 5% was assumed at the inlet. Flow rate curves at the MPA were generated synthetically using a hybrid approach. First, cardiac outputs (CO) and heart rates (HR) were estimated (see Table 1) based on the animals’ weights, according to scaling laws for pigs (18). Next, flow rates were synthetically generated based on principal component analysis of MRI-measured MPA flow waveforms in pigs, which were published earlier (19). Figure 3 shows all 10 simulated MPA flow rate waveforms with different heart rates, stroke volumes and curve shapes including different peak systolic volume flow rates. Pre- and post-treatment simulations were performed using the same boundary conditions.

**Table 1:** Overview of the animal weights, pulmonary artery (PA) surface areas, as well as hemodynamic baseline characteristics used for specification of the inflow boundary conditions.

Animal	Weight (kg)	HR (bpm)	CO (L/min)	$Q_{\max}$ (mL/s)	PA area (cm${}^{2}$)
Case 01	62	99	4.7	245	135
Case 02	59	101	4.6	224	156
Case 03	58	102	4.6	216	169
Case 04	72	95	5.1	220	136
Case 05	55	104	4.4	238	138
Case 06	65	104	4.4	241	164
Case 07	65	98	4.8	176	150
Case 08	65	98	4.9	238	149
Case 09	65	98	4.8	173	149
Case 10	65	98	4.8	189	171

HR, heart rate; CO, cardiac output; $Q_{\max}$, peak systolic volume flow rate.

Table 1 summarizes demographic and hemodynamic information of the animals as well as the inflow boundary conditions used for the CFD simulations.

### 2.5. CFD results post-processing

Simulation results were post-processed using Matlab R2021a (MathWorks, Natick, MA, USA). Three parameters were evaluated: TAWSS, OSI, and static pressure. TAWSS computes the temporal mean of local WSS as defined in the Equation 1:

(1) $$\mathrm{TAWSS}=\frac{1}{N}\sum_{t=1}^{N}\sqrt{{\mathrm{WSS}}_{Xt}^{2}+{\mathrm{WSS}}_{Yt}^{2}+{\mathrm{WSS}}_{Zt}^{2}}$$

where ${\mathrm{WSS}}_{Xt}$ is the local WSS in x-direction at a time step t and N is the number of equidistant time steps describing the whole heart cycle. OSI is a measure of directional change of the WSS over time ranged between 0 and 0.5. It is defined in the Equation 2:

(2) $$\begin{array}{r} \mathrm{OSI}=\frac{1}{2}\left(\;\;1-\frac{\sqrt{(\sum_{t=1}^{N}{\mathrm{WSS}}_{Xt}{)}^{2}+(\sum_{t=1}^{N}{\mathrm{WSS}}_{Yt}{)}^{2}+(\sum_{t=1}^{N}{\mathrm{WSS}}_{Zt}{)}^{2}}}{\sum_{t=1}^{N}\sqrt{{\mathrm{WSS}}_{Xt}^{2}+{\mathrm{WSS}}_{Yt}^{2}+{\mathrm{WSS}}_{Zt}^{2}}}\right) \end{array}$$

whereas no direction change of the WSS vector over time means that the value for OSI becomes zero while a harmonic oscillation of the WSS vector results in the OSI of 0.5. Beside surface averaged values for the TAWSS and OSI, we also calculated areas with low WSS (WSS<0.5 Pa) as well as areas with high OSI (OSI>0.2). Finally the pressure drop caused by the sensor was calculated as a difference between cross-section averaged static pressure measured 10 mm downstream and 10 mm upstream of the sensor. Statistical analysis was performed using IBM SPSS Statistics 28 (IBM, USA). Mean and standard deviation were reported for normally distributed parameters. Normality of distribution was assessed using a Shapiro-Wilk test. For non-normally distributed parameters, median and interquartile [IQR] range were used to report parameter distributions. All tests used a standard significance level of 0.05.

## 3. Results

### 3.1. Pulmonary artery geometry before device implantation

**Figure 4:**
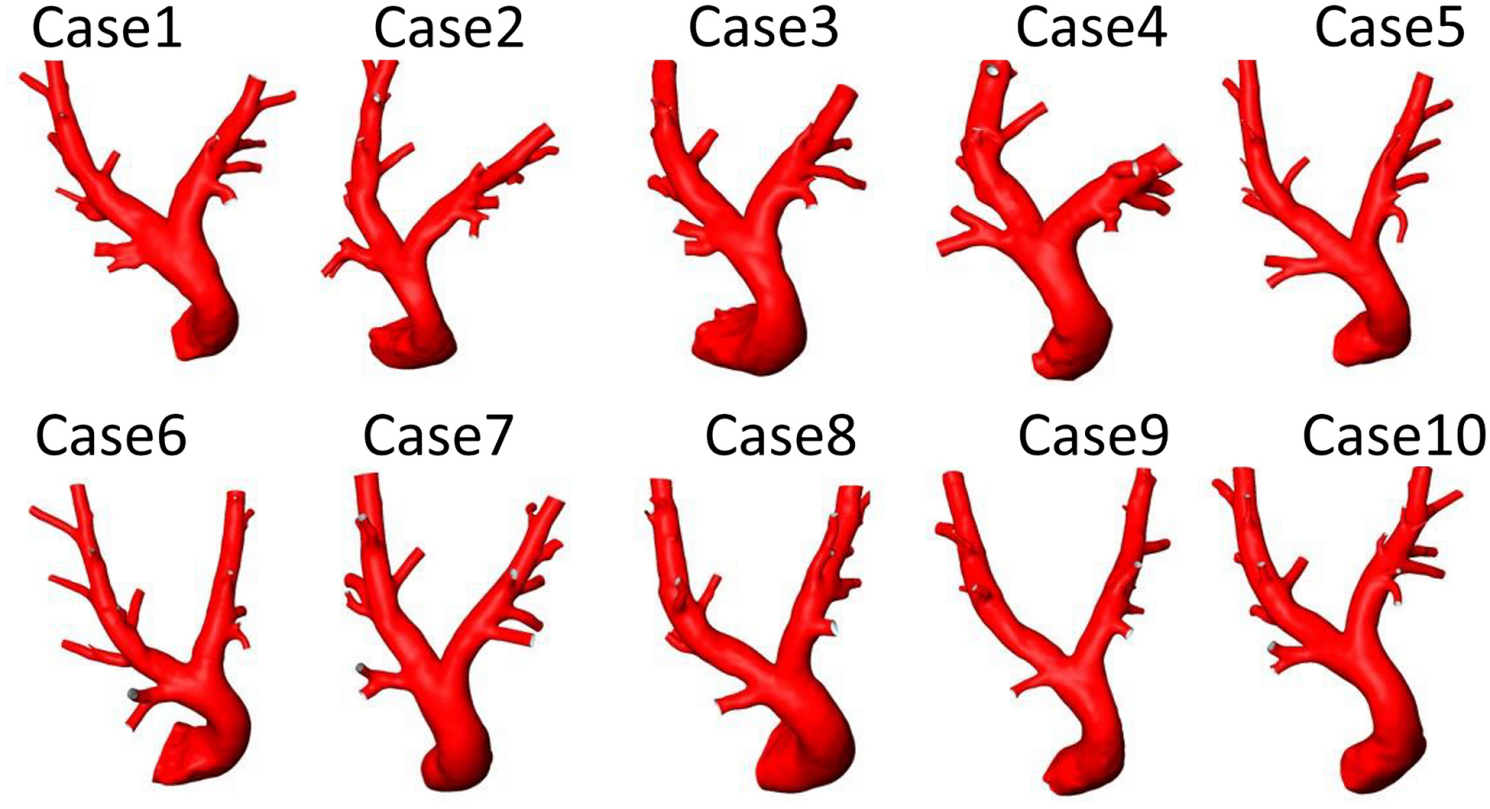
Geometries of 10 porcine pulmonary arteries reconstructed from computed tomography data acquired before the pulmonary artery pressure sensor implantation.

The geometries of all 10 porcine PA reconstructed from the pre-interventional CT images are shown in Figure 4. All geometric parameters evaluated for these geometries are summarised in Table 2. The average lengths of the MPA, RPA, and LPA were 66.5±11.2 mm, 114.1±14.3 mm, and 101.9±12.2 mm, respectively. The average and standard deviations of these vessels’ diameters were 23.8±2.2 mm, 14.9±2.0 mm, and 13.6±1.7 mm. The bifurcation angle between LPA and RPA was on average 80±7 degrees. Average and standard deviations of the number of side branches were 7±1.3 and 6±1.1 in the LPA and RPA, respectively. RPA length was not significantly different then the LPA (p=0.059, Wilcoxon test), however the RPA diameter was significantly larger then the LPA diameter (p=0.047, Wilcoxon test).

**Table 2:** Overview of all individual values for the lengths (L) and diameters (D) of the main (MPA), left (LPA), and right pulmonary artery (RPA), as well as the bifurcation angle (α) between LPA and RPA.

Animal	MPA L (mm)	MPA D (mm)	RPA L (mm)	RPA D (mm)	LPA L (mm)	LPA D (mm)	α (${}^{\circ }$)
Case 01	59.4	21.4	115.5	13.2	100.9	12.1	81
Case 02	78.0	21.3	115.1	13.1	105.4	12.7	73
Case 03	82.7	28.0	105.9	15.5	91.6	14.9	85
Case 04	52.0	23.3	81.1	15.3	105.9	17.1	91
Case 05	56.2	22.2	122.9	13.0	103.1	12.6	84
Case 06	76.6	24.3	129.6	12.1	102.4	12.1	86
Case 07	56.9	25.6	106.0	17.5	114.4	12.5	79
Case 08	69.6	26.0	113.8	16.7	103.2	12.8	75
Case 09	58.1	22.5	130.0	17.5	117.9	15.2	68
Case 10	75.8	24.0	121.2	14.6	73.9	13.5	82

### 3.2. Analysis of device position

**Figure 5:**
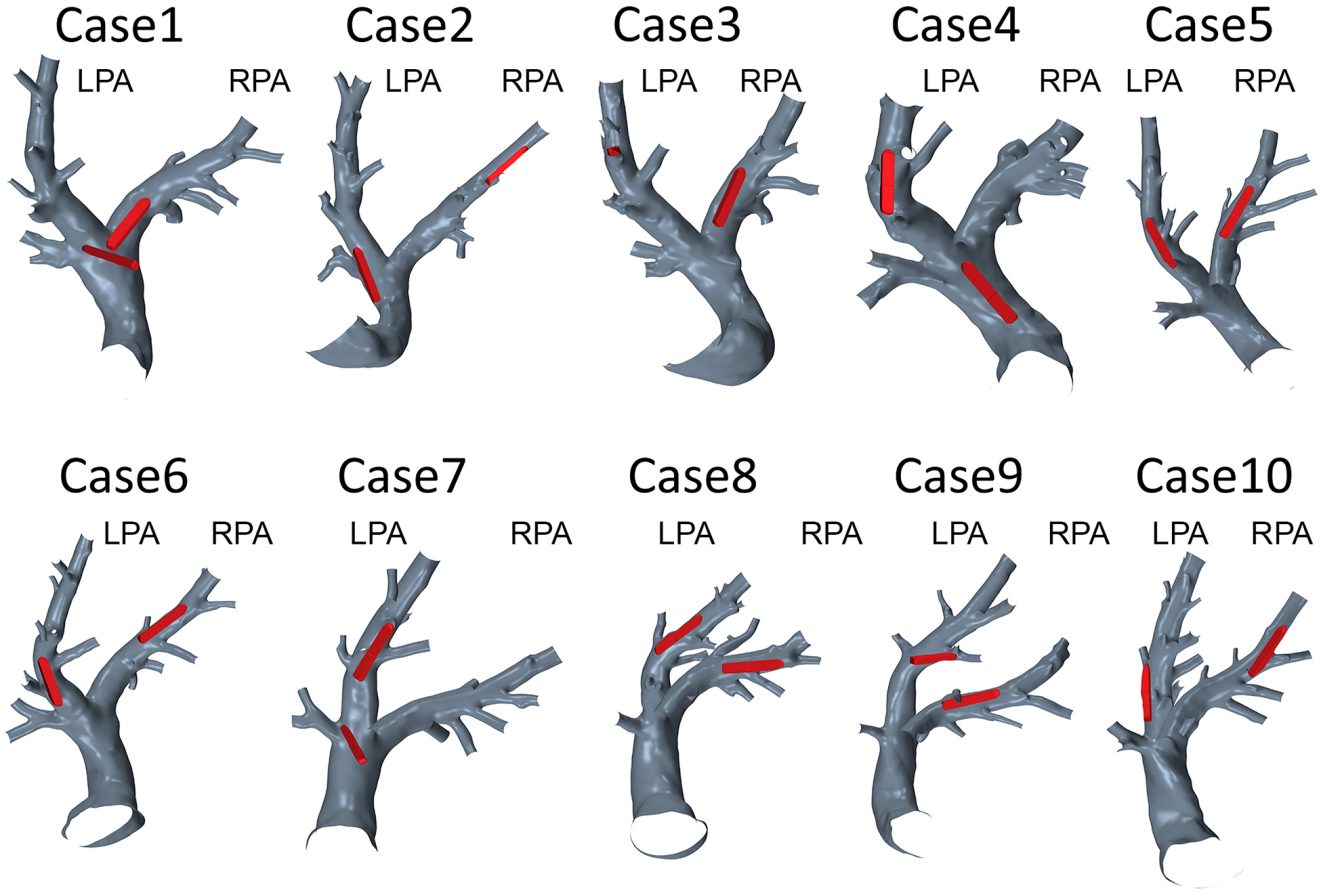
Geometries of 10 pulmonary arteries together with the respective device positions in the left (LPA) or right (RPA) pulmonary artery.

**Figure 6:**
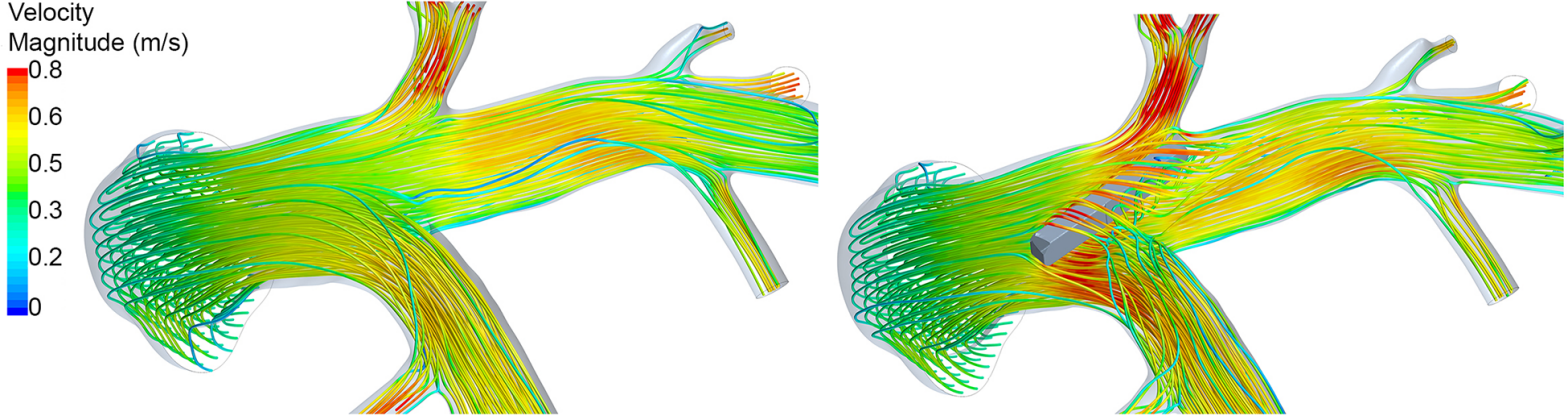
Illustration of the peak-systolic hemodynamics using streamlines in Case 07 before (left) and after (right) virtual device implantation. Streamlines are color-coded using the velocity magnitude. After device implantation, marked perturbation of the flow is observed due to the non-optimal device position.

The implantation sites of all 20 devices were analyzed with respect to the device position and the vessel diameter. In 12 cases, the device was located in an optimal position regarding hemodynamics. The remaining 8 cases, i.e., both devices in Cases 1 and 3, the RPA devices in Cases 5, 6, and 9, as well as a sensor located in the MPA in redCase 7, were considered to be non-optimal. The position of all devices within the respective PA anatomy are illustrated in Figure 5. Optimal implantation was present, when the entire device is pressed against the vessel wall, causing a minimal flow disturbance and blocking of the vessel. In contrast, a sub-optimal implantation was present, when the device was skewed, reaching from one vessel wall to the other, was located within a side branch (e.g., in Case 3), or was covering a side branch. An example of a skewed device and the resulting hemodynamics is illustrated in Figure 6. In addition to this classification in optimal and non-optimal position, three devices (each one in Cases 1, 4, and 7) were located in the MPA instead of the LPA or RPA, meaning that the vessel diameter at the final implantation site was much larger than the recommended one.

### 3.3. Pulmonary artery hemodynamics before device implantation

**Figure 7:**
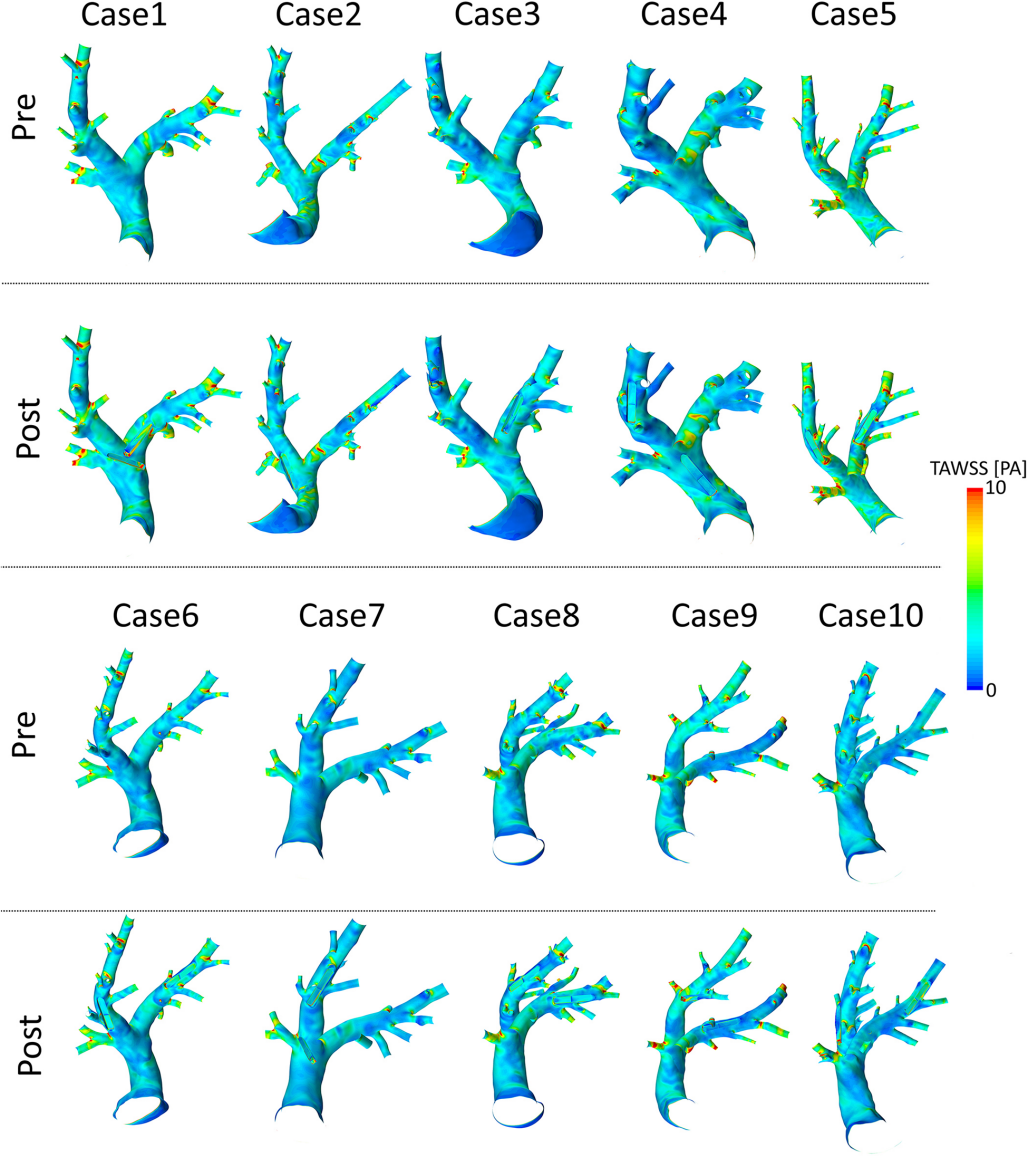
Spatial distributions of time-averaged wall shear stress (TAWSS) values before (pre) and after (post) virtual device implantation.

**Figure 8:**
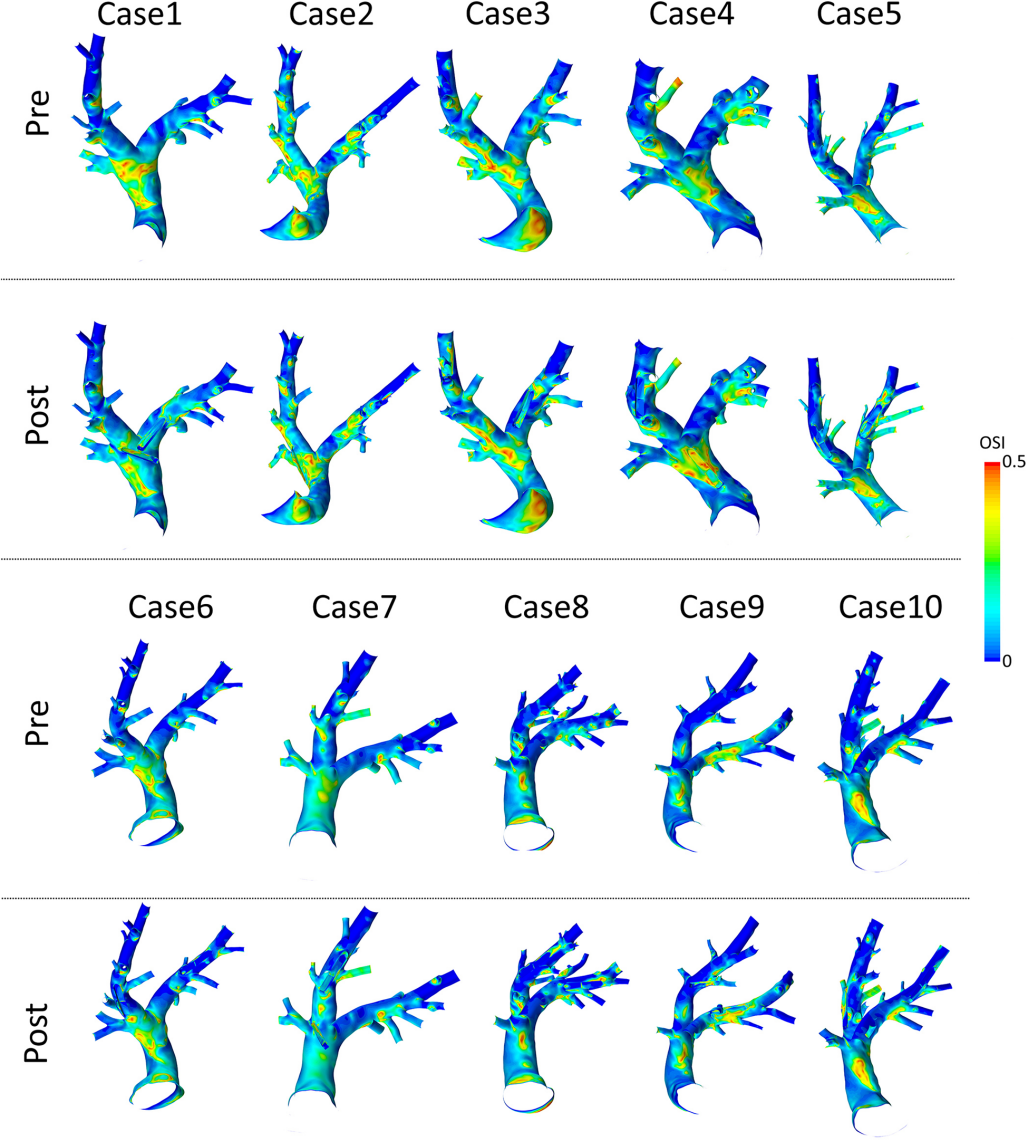
Spatial distributions of oscillatory shear index (OSI) values before (pre) and after (post) virtual device implantation.

Spatial distributions of TAWSS and OSI for all 10 pre-interventional geometries are shown in Figures 7 and 8, respectively. The averaged surface-averaged TAWSS was 2.35±0.47 Pa, whereas the surface-averaged OSI was 0.08±0.17. Areas affected by low WSS magnitudes were on average 2.5±2.7 cm${}^{2}$, whereas the areas affected by high OSI were 18.1±6.3 cm${}^{2}$. The overall surface area of the PA geometries was on average 152.0±13.2 cm${}^{2}$. The individual values for TAWSS, OSI, as well as the corresponding surface areas are provided in Table 3.

**Table 3:** Overview of all individual parameters describing intra-arterial hemodynamics before and after sensor implantation.

Animal	WSS (Pa)	WSS (Pa)	OSI (-)	OSI (-)	lWSSa (cm${}^{2}$)	lWSSa (cm${}^{2}$)	hOSIa (cm${}^{2}$)	hOSIa (cm${}^{2}$)
	Pre	Post	Pre	Post	Pre	Post	Pre	Post
Case 01	2.71	2.96	0.07	0.08	0.02	0.18	14.46	15.08
Case 02	2.63	2.70	0.09	0.09	0.42	1.01	21.60	23.40
Case 03	1.86	1.96	0.11	0.11	7.92	8.52	32.40	31.29
Case 04	2.22	2.30	0.09	0.10	0.92	1.64	23.63	22.59
Case 05	3.37	3.48	0.09	0.09	0.24	1.04	16.48	17.92
Case 06	2.14	2.23	0.07	0.07	2.99	3.37	15.21	16.04
Case 07	1.89	2.00	0.07	0.07	1.06	1.39	12.38	11.13
Case 08	2.22	2.23	0.08	0.08	6.55	7.07	17.95	18.23
Case 09	2.56	2.70	0.06	0.06	1.51	1.69	12.28	11.36
Case 10	1.92	1.98	0.06	0.07	2.94	3.46	14.36	16.84

WSS, surface- and time-averaged WSS; OSI, surface-averaged OSI; lWSSa, pulmonary arterey surface area affected by low (<0.5 Pa) WSS; hOSIa, pulmonary artery surface area affected by high (>0.2) OSI.

### 3.4. Pulmonary artery hemodynamics after device implantation

Spatial distributions of TAWSS and OSI for all 10 geometries after virtual device implantation are shown in the lower rows of Figures 7 and 8, respectively. As the devices reduce the cross-section of the lumen and thus act as an obstacle, they might cause flow disturbances resulting in changes of WSS and OSI. On average, TAWSS and OSI after device implantation were 2.45±0.49 Pa and 0.08±0.16, respectively. While both TAWSS and OSI were significantly larger after device implantion compared to the pre-interventional state (p=0.001 and p<0.001, paired Student’s t-test) the respective effect sizes of these changes were 0.22 and 0.01 and are therefore not relevant. In addition to these parameters that are associated with thrombus formation, the static pressure drop across the vessel segments in which the devices were implanted were quantified. On average, the pressure drop was 0.7±1.1 mmHg. In some cases, calculated pressure drops are negative, indicating an increase in static pressure. This is caused by an increase in the cross-sectional area of the PA across the device. Finally, the surface areas affected by low TAWSS and high OSI after sensor implantation were 2.9±2.7 cm${}^{2}$, and 18.4±6.1 cm${}^{2}$, respectively. A significant increase in the surface area affected by low TAWSS (p=0.005, Wilcoxon signed rank test), was observed, however the absolute different was only 0.48±0.22 cm${}^{2}$, which roughly equals 0.3% of the overall PA surface, and is therefore negligible. All individual parameters are provided in Table 3.

In addition to the hemodynamics at the PA surfaces, the device bodies were investigated separately. TAWSS averaged over all 20 devices was 3.07±0.89 Pa and therefore slightly larger than the values observed at the vessel wall, while still being in the same range. No significant difference for TAWSS values at the device surface was found between sensors implanted in the LPA and RPA (3.13±0.93 Pa vs. 3.00±0.90 Pa, p=0.707, paired Student’s t-test). The difference in TAWSS for PAPS and PA surfaces were significant for sensors implanted in the LPA and the RPA (p=0.05 and (p=0.035, respectively, paired Student’s t-test). Similarly, OSI calculated at the device surface was 0.11±0.06 and thus slightly larger than the averages at the vessel wall. However in contrast to the TAWSS, differences for OSI between PAPS and PA surfaces were not significant for LPA and RPA (paired t-Student test). Furthermore, no significant difference for OSI values at the device surface was found between sensors implanted in the LPA and RPA (0.11±0.07 vs. 0.11±0.06, p=0.964, paired Student’s t-test). The individual values for OSI, TAWSS, and the pressure drop for all 20 devices are provided in Table 4.

**Table 4:** Overview of all individual parameters describing device-related hemodynamics.

Animal	WSS (Pa)	WSS (Pa)	OSI (-)	OSI (-)	lWSSa (cm${}^{2}$)	lWSSa (cm${}^{2}$)	hOSIa (cm${}^{2}$)	hOSIa (cm${}^{2}$)	dP (mmHg)	dP (mmHg)
	PAPS I	PAPS II	PAPS I	PAPS II	PAPS I	PAPS II	PAPS I	PAPS II		
Case 01	4.88	4.38	0.08	0.13	0.08	0.07	0.48	1.26	1.0	0.4
Case 02	3.15	2.78	0.04	0.24	0.22	0.06	0.18	1.77	2.2	1.6
Case 03	3.59	3.65	0.07	0.05	0.08	0.25	0.38	0.18	0.7	−0.8
Case 04	2.37	2.08	0.25	0.08	0.10	0.42	1.55	0.43	−0.5	−0.5
Case 05	3.70	3.80	0.12	0.10	0.10	0.25	0.62	0.43	1.0	0.2
Case 06	2.98	2.43	0.05	0.08	0.18	0.33	0.26	0.50	2.9	0.1
Case 07	3.89	2.19	0.09	0.11	0.04	0.25	0.70	0.49	0.4	0.4
Case 08	2.25	2.27	0.11	0.04	0.34	0.27	0.67	0.17	2.8	1.4
Case 09	1.64	4.16	0.21	0.12	0.19	0.21	1.19	1.11	−0.3	1.1
Case 10	2.89	2.26	0.04	0.13	0.32	0.35	0.19	0.93	0.3	−0.7

WSS, surface- and time-averaged WSS; OSI, surface-averaged OSI; lWSSa, PA surface with low (<0.5 Pa) WSS; hOSIa, PA area with high (>0.2) OSI; PAPS I, sensor in LPA; PAPS II, sensor in RPA.

Finally, the surface areas of the device affected by low TAWSS and high OSI were analyzed. The average device area with low WSS was $0.2\pm 0.1\;{\mathrm{cm}}^{2}$ and therefore 10 times smaller than the area with low WSS measured at the PA surface. In relative measures, less than 7 % of the device surface were affected by low WSS. The device surface affected by high OSI was with median of $0.49\;{\mathrm{cm}}^{2}$ and IQR of $[0.29-1.06]\;{\mathrm{cm}}^{2}$ more than 30 times smaller compared to the vessel area affected by high OSI. In relative measures, less than 17 % of the device surfaces were affected by high OSI.

### 3.5. Hemodynamics of optimal vs. non-optimal PAPS implantations

To compare, whether non-optimal device position results in hemodynamic differences, the above-mentioned parameters were also compared between the 12 optimally and 8 non-optimally positioned devices. First, the device area in contact with blood was significantly larger in the non-optimally positioned devices (3.8±0.6 cm${}^{2}$ vs. 2.7±0.3 cm${}^{2}$ vs. p=0.001, Student’s t-test). TAWSS at the device surface was significantly smaller in the optimal compared to the non-optimal cases (2.55±0.56 Pa vs. 3.85±0.71 Pa, p<0.001, Student’s t-test). No significant differences were found for OSI at the device surface (0.12±0.08 vs. 0.09±0.03, p=0.263). Similarly, the comparison of areas with low WSS and high OSI showed no significant differences between optimally and non-optimally implanted PAPS. No differences were found for the device area affected by low WSS (0.23±0.11 cm${}^{2}$ vs. 0.16±0.11 cm${}^{2}$, p=0.188, Student’s t-test) or high OSI (0.70±0.55 cm${}^{2}$ vs. 0.63±0.37 cm${}^{2}$, p=0.744, Student’s t-test). Finally no significant difference was found between pressure drop caused by optimally and non-optimally positioned devices (0.9±1.3 mmHg vs. 0.4±0.6 mmHg, p=0.264, Student’s t-test).

## 4. Discussion

In-silico studies are common in research of pathologies affecting the pulmonary artery, such as pulmonary stenosis and pulmonary hypertension (20–22). Numerical assessment of medical devices, either by investigating implantation procedures using finite element modelling or hemodynamic device efficacy using CFD is also common (23–25). However, so far no studies investigating the effects of PAPS devices on the intra-arterial hemodynamics were published for either of the existing systems as indicated by a literature research in PubMed using the terms “pulmonary artery pressure sensor,” “in-silico,” “Cordella,” “CardioMEMS,” “CFD,” “hemodynamics” on 1st of March 2023.

In this study, we were able to enhance an in-vivo animal experiment by CFD-based calculation of the intra-arterial hemodynamics. Thus, a set of parameters associated with thrombus formation and vascular remodelling, namely WSS and OSI, could be calculated for each animal investigated within the experiments. More importantly, hemodynamics have been calculated before and after implantation of the device, while mimicking the exact device position as closely as possible using spatially well resolved CT imaging. This in-silico analysis of the intra-arterial hemodynamics revealed no relevant changes in any hemodynamic parameters due to the sensor implantation. Note, that device implantation was not only performed for the optimal, but also for sub-optimal positions such as skewed across the arterial cross-section, located in the MPA or in small side branches. This was done to assess the device function in a wider range of configurations and yield a broader parameter distribution for validation of the models and increase the chance of occurrence of device thrombosis. However, even in cases, where devices were implanted in a non-optimal manner, no marked differences in any investigated hemodynamic parameters compared to devices implanted in their intended site and orientation were found.

This finding agrees well with the outcomes of our animal experiments. The chronic experiments ran for approximately 60 days after sensor implantation. None of the animals showed any symptoms of lung embolism. Furthermore, after explantation of the devices and euthanasia of the animals, the lungs were extracted and evaluated for any signs of embolisms, which were not present in any of the animals. While these findings might be considered favorable with respect to device efficacy and safety, the aim of the animal experiments was to provide data elements for validation of numerical models for prediction of clinical relevant outcomes, such as thrombosis. As no embolic events have been observed in any of the animals, we were not able to validate, that these events or at least the increased risk for their occurrence can be predicted using the proposed in-silico approach.

However, the evaluated hemodynamic parameters are generally accepted to be strongly associated with thrombus formation (26, 27) and also have been used in similar investigations focusing on device efficacy and safety of implantable cardiovascular devices (15). In addition, incidence rates for embolic events reported in relevant PAPS trials are also very low, ranging from 0 to 1% (28). Therefore, the non-occurence of any embolic events within the limited duration of the animal experiments is in line with these reports.

Furthermore, the in-silico modelling accompanying the study allowed to assess the hemodynamic changes caused by the implanted devices in detail. This information could otherwise not be acquired using in-vivo experiments. Using the simulations, the initial hypothesis, that an non-optimal sensor position will result in significantly increased flow disturbances, measured by changes in WSS and OSI, and therefore higher risks of embolic events, could be falsified.

This study highlights the strong benefits from including in-silico studies in animal experiments for assessing device effect, efficacy, and safety. This approach provides additional information to better understand the results of the animal experiments, gain more insights from them, and enhance the available parameters in a relevant manner. Therefore, in-silico modelling is a viable way to address the “refine” aspect embedded in the 3R principle. In addition, mimicking animal experiments and human trials using in-silico approaches is an ideal way to provide evidence for the applicability of these methods. If the in-silico methods are able to predict clinical outcomes in a sufficient manner, they might even be able to reduce animal experiments or replace them altogether.

### 4.1. Limitations

This study is associated with some limitations that should be noted. First, flow rate waveforms used as inlet boundary conditions were generated synthetically, as no subject-specific measurements were available. Second, simulation were performed assuming rigid walls, neglecting the vessel distensibility. Studies using fluid structure interaction to asses hemodynamics in healthy PA reported overestimated WSS when using rigid walls (29). However, PAPS are used in HF patients, which are associated with significantly stiffer PA (30). Furthermore, the recently published work of Kong et al. regarding fluid-structure interaction (FSI) simulations of the PA tree reported only slight differences in wall shear stress calculated using FSI compared against simulations with rigid walls (22). As the main focus of this study was assessing the hemodynamic changes caused by the device, the effects of these assumptions are assumed to be minimal.

Additionally, two sensors were implanted in each animal to reduce the sample size, adhering to the 3R principles. The intended use for the sensor, however, is only one sensor to be implanted into one side of the pulmonary artery. As the pressure gradients across the sensor were approximately 1.0 mmHg and therefore are smaller than the PA pressure, no relevant change in resistance and therefore in flow distribution is to be expected. Implantation of a second device might result in dislocation of the first device.

In this study, only OSI and TAWSS were calculated to assess thrombosis risk as well as occurence of flow disturbances, even though a large number of potential parameters are discussed. These parameters have been selected as they are the most commonly used parameters, common thresholds for them are suggested, and they are applied to a wide range of applications, such as abdominal aortic aneurysms (31, 32), left atrial appendage thrombus formation (33), and medical device thrombosis (34). Another commonly evaluated parameter, the relative residence time (RRT) can be calculated from TAWSS and OSI and was therefore considered redundant for the purpose of this study. Additionally, only wall-bound parameters but no intra-vascular hemodynamics, such as shear rates and recirculation regions were investigated.

Finally, the chronic animal experiment were only run for three months and thrombosis is known to be a long-term effect. However, acute thrombosis, which occurs within 24 h of initial placement as well as subacute or earlier thrombosis, which occurs between 24 h to one month of initial placement are assessed in our animal study. Thus, chances for thrombus formation might have increased with longer study duration. These however, were not possible due to constraints arising from the animals’ growth.

## 5. Conclusion

In this study we were able to enhance in-vivo animal experiments using in-silico models, mimicking the interventions performed. Using this approach, additional hemodynamic parameters, which cannot be acquired in-vivo, could be made available. Based on both the results of the in-vivo and in-silico study, no relevant differences in hemodynamics after sensor implantation are expected. Even at non-optimal positioning of the sensor, neither significant changes in hemodynamics, nor embolic events could be observed, suggesting that risk for thrombus formation due to hemodynamic alterations is low independent of the sensor position. However, this hypothesis has to be further evaluated in device thrombogenicity studies, following relevant standards, such as ISO 10993-4 norm. Finally, the second aim of the study to validate the risk of thrombus formation based on hemodynamic simulations could not be validated due to the non-occurence of any embolic events. While longer running experiments with larger sample sizes might increase the risk for occurence of these events, they would come at a high ethical burden.

## Data Availability

The datasets presented in this study can be found in online repositories. The names of the repository/repositories and accession number(s) can be found below: FigShare, 10.6084/m9.figshare.22263016.
