# Supplementary material for: In-silico enhanced animal study of pulmonary artery pressure sensors: assessing hemodynamics using computational fluid dynamics
Source: Front Cardiovasc Med. 2023 Sep 7;10:1193209. doi: 10.3389/fcvm.2023.1193209 (PMC10517052; doi:10.3389/fcvm.2023.1193209)
Supplement: Supplementary file 1 [file Datasheet1.pdf]

**Mesh Sensitivity Analysis**

A mesh sensitivity analysis was performed to assess the sensitivity of the hemodynamic parameters evaluated in this study to the chosen mesh resolution. Here, only the parameter 'base size' was adjusted for the different mesh resolutions. The thickness of the prism layers at the vessel wall have been specified as relative value of the base size and were therefore not manually modified. All other mesh parameters, such as the number of prism layers were not adjusted. The following base size values were investigated: 1.25 mm, 1.00 mm, 0.75 mm, 0.50 mm. The mesh sensitivity analysis was performed for two cases. For both cases the oscillating shear index (OSI) and the time-averaged wall shear stress (TAWSS) were evaluated. The results of the mesh sensitivity analysis are shown in the table below.

| | base size | 1.25 mm | 1.00 mm | 0.75 mm | 0.50 mm |
|---|---|---|---|---|---|
| **Case #1** | OSI absolute [-] | 0.063 | 0.063 | 0.060 | 0.062 |
| | OSI relative [%] | 102.5 | 101.4 | 96.8 | 100.0 |
| | TAWSS absolute [Pa] | 2.52 | 2.54 | 2.56 | 2.62 |
| | TAWSS relative [%] | 96.1 | 96.8 | 97.6 | 100 |
| **Case #2** | OSI absolute [-] | 2.68 | 2.68 | 2.74 | 2.81 |
| | OSI relative [%] | 95.3 | 95.5 | 97.6 | 100.0 |
| | TAWSS absolute [Pa] | 0.073 | 0.071 | 0.069 | 0.070 |
| | TAWSS relative [%] | 104.9 | 101.8 | 98.7 | 100.0 |

Supplemental Table 1: *Absolute and relative values of oscillatory shear index (OSI) and time-averaged wall shear stress (TAWSS) for two cases calculated for different mesh sizes. Please note, the case IDs are not equal to the case IDs in the manuscript.*

Deviations in the surface averaged values between the different mesh sizes were small. Therefore, the mesh size of 0.75 mm was chosen as being sufficient for the simulations to be performed within this study. To also assess the similarity of the distributions of both OSI and TAWSS, these field werde exported as .csv file. The results obtained using the finer mesh of 0.50 mm base size were interpolated onto the mesh with a base size 0f 0.75 mm, using the *scatteredInterpolant* function provided by MATLAB. Subsequently, the correlation coefficient between the absolute values of OSI and TAWSS calculated using these two base sizes were calculated. For both cases and both parameters investigated, the coefficient of determination was $R^2 > 0.99$. This indicates that the chosen mesh size is sufficient to provide reliable results not affected by the spatial discretization.

**Periodic Convergence Analysis**

Similar to the mesh sensitivity analysis, a periodic convergence analysis was performed. In numerical assessment of periodic flows, the periodic convergence is important, as simulations usually start with a non-physiological initial condition, which is that the flow velocities in each part of the artery are zero. Therefore, the first cycle calculated using CFD is usually not yet converged. However, even for the subsequent cycles, residual effects of this initial condition can be maintained. Therefore, a periodic convergence analysis is carried out to assess whether the parameters evaluated within the simulation differ between the different heart cycles. For this, simulations run for a total of 5 heart cycles. Then, the same parameters as for the mesh sensitivity analysis were evaluated for the different cycles. The results are shown in the table below.

| | Heart | 2 | 3 | 4 | 5 |
|---|---|---|---|---|---|
| **Case #1** | OSI absolute [-] | 2.6 | 2.6 | 2.6 | 2.6 |
| | OSI relative [%] | 100.3 | 100.1 | 100.1 | 100.0 |
| | TAWSS absolute [Pa] | 0.061 | 0.061 | 0.060 | 0.060 |
| | TAWSS relative [%] | 101.6 | 101.4 | 100.8 | 100.0 |

| | | | | | |
|---|---|---|---|---|---|
| Case #2 | OSI absolute [-] | 2.74 | 2.74 | 2.74 | 2.74 |
| | OSI relative [%] | 100.0 | 100.0 | 100.0 | 100.0 |
| | TAWSS absolute [Pa] | 0.069 | 0.069 | 0.069 | 0.069 |
| | TAWSS relative [%] | 100.1 | 100.0 | 100.0 | 100.0 |

Supplemental Table 2: *Absolute and relative values of oscillatory shear index (OSI) and time-averaged wall shear stress (TAWSS) for two cases calculated during different consecutive heart cycles. Please note, the case IDs are not equal to the case IDs in the manuscript.*

There are no relevant differences observed in the cycle-averaged parameters reported in this study between running the simulation for 2 or up to 5 consecutive heart cycles. Therefore, evaluating the second heart cycle was considered to be entirely sufficient for this study. In addition, the area percentages during peak-systole being affected by either high or low WSS were matching perfectly as well (differences less than 0.2 %), indicating that even at a non-cycle-averaged evaluation the solution is already converged during the second heart cycle.

**Operator Bias Analysis**

The reconstruction procedure performed in this study mostly relied on manual labelling. Therefore, we assessed the accuracy of this reconstruction procedure by performing an operator bias analysis. Here, the pulmonary artery anatomy was reconstructed from ten computed tomography datasets by two independent operators. This analysis was performed using image data obtained from patients and not from pigs. However, the same imaging sequences were used.

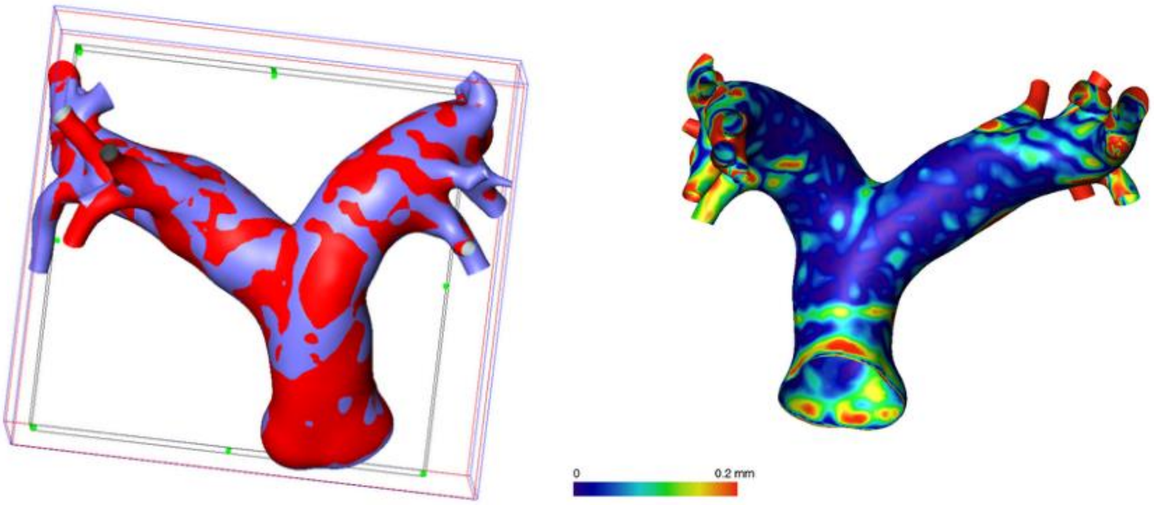


Supplemental Figure 1: *Overlay of two manual reconstructions of the same pulmonary artery (left). Visualization of the surface distances between the two reconstructions (right). Here, a color threshold of the approximate voxel resolution (0.2 mm) was used.*

Very good agreement with respect to the main, left and right pulmonary artery reconstructed by the different operators was observed (see Supplemental Figure 1). The average deviation between the surfaces reconstructed by the two operators was below 0.2 mm and therefore below the image resolution of the CT data. Large deviations were only observed for the branching vessels as here manual reconstructions resulted in different lengths of the branching vessels.
